# Racial disparities in colorectal cancer outcomes and access to care: a multi-cohort analysis

**DOI:** 10.3389/fpubh.2024.1414361

**Published:** 2024-06-19

**Authors:** Paul Riviere, Kylie M. Morgan, Leah N. Deshler, Joshua Demb, Winta T. Mehtsun, Maria Elena Martinez, Samir Gupta, Matthew Banegas, James D. Murphy, Brent S. Rose

**Affiliations:** ^1^Department of Radiation Medicine and Applied Sciences, University of California, San Diego, La Jolla, CA, United States; ^2^Center for Health Equity Education & Research (CHEER), University of California, San Diego, La Jolla, CA, United States; ^3^Veterans Affairs San Diego, La Jolla, CA, United States; ^4^Division of Gastroenterology, Department of Medicine, University of California, San Diego, La Jolla, CA, United States; ^5^Division of Surgical Oncology, Department of Surgery, University of California, San Diego, La Jolla, CA, United States; ^6^Division of Preventive Medicine, Department of Family Medicine and Public Health, University of California, San Diego, La Jolla, CA, United States

**Keywords:** colorectal cancer, disparities, health services research, outcomes, race, veteran affairs

## Abstract

**Introduction:**

Non-Hispanic Black (NHB) Americans have a higher incidence of colorectal cancer (CRC) and worse survival than non-Hispanic white (NHW) Americans, but the relative contributions of biological versus access to care remain poorly characterized. This study used two nationwide cohorts in different healthcare contexts to study health system effects on this disparity.

**Methods:**

We used data from the Surveillance, Epidemiology, and End Results (SEER) registry as well as the United States Veterans Health Administration (VA) to identify adults diagnosed with colorectal cancer between 2010 and 2020 who identified as non-Hispanic Black (NHB) or non-Hispanic white (NHW). Stratified survival analyses were performed using a primary endpoint of overall survival, and sensitivity analyses were performed using cancer-specific survival.

**Results:**

We identified 263,893 CRC patients in the SEER registry (36,662 (14%) NHB; 226,271 (86%) NHW) and 24,375 VA patients (4,860 (20%) NHB; 19,515 (80%) NHW). In the SEER registry, NHB patients had worse OS than NHW patients: median OS of 57 months (95% confidence interval (CI) 55–58) versus 72 months (95% CI 71–73) (hazard ratio (HR) 1.14, 95% CI 1.12–1.15, *p* = 0.001). In contrast, VA NHB median OS was 65 months (95% CI 62–69) versus NHW 69 months (95% CI 97–71) (HR 1.02, 95% CI 0.98–1.07, *p* = 0.375). There was significant interaction in the SEER registry between race and Medicare age eligibility (*p* < 0.001); NHB race had more effect in patients <65 years old (HR 1.44, 95% CI 1.39–1.49, p < 0.001) than in those ≥65 (HR 1.13, 95% CI 1.11–1.15, p < 0.001). In the VA, age stratification was not significant (*p* = 0.21).

**Discussion:**

Racial disparities in CRC survival in the general US population are significantly attenuated in Medicare-aged patients. This pattern is not present in the VA, suggesting that access to care may be an important component of racial disparities in this disease.

## Introduction

Colorectal cancer is the second-leading cause of cancer mortality in the United States (1) and is a disease characterized by significant racial disparities. Specifically, non-Hispanic Black (NHB) Americans, along with Alaskan Native/American Indian individuals (2), have among the highest risks of developing colorectal cancer (CRC) of any racial group in the United States (3), almost 25% higher than in non-Hispanic white (NHW) Americans (1). Even as the mortality rate nationwide has consistently decreased for the NHW population, these improvements have been less pronounced in NHB women, whereas NHB men have had a consistent increase in mortality (4). From 2014 to 2018, NHB adults had more than a 30% increased mortality compared to NHW adults (5). Among those diagnosed with CRC, NHB patients have worse stage-specific survival (4) and are more likely to have advanced disease at diagnosis (1).

Inequalities in access to care likely contribute to these observed survival disparities. First, NHB patients were historically less likely than NHW patients to receive screening colonoscopies (6, 7), which can both improve cancer mortality through earlier detection and decrease cancer incidence via removal of pre-malignant lesions; (8, 9) however, more recent nationwide data suggest that this gap has closed (10). Second, in the setting of randomized clinical trials with standardized treatment (usually delivered in specialized centers), NHB patients appear to have equivalent survival rates compared to their NHW peers (11). Third, studies based on cancer registry data suggest that NHB patients are less likely to receive evidence-based treatments once diagnosed with CRC (12). These latter two points are especially important when considering the increases in early-onset colorectal cancer (13, 14), which is typically not detected through screening.

We sought to quantify the relative overall survival following CRC diagnosis in NHB and NHW individuals in two cohorts, one from a nationwide cancer registry, and one from the United States Veterans Health Administration (VA), in which patients have equal access to care. Additionally, analyses were stratified based on age ≥ 65 to account for the effect of Medicare age eligibility.

## Materials and methods

### Patient population

Two separate cohorts of colorectal cancer patients were assembled, one from the VA and the second from the Surveillance, Epidemiology, and End Results (SEER) database. The VA cohort was assembled through the VA Informatics and Computing Infrastructure (VINCI), which contains the VA cancer registry, medical claims data, and diagnostic results of all patients treated in the VA healthcare system. The SEER cohort was accessed through SEER*Stat (15). The SEER is a nationwide cancer registry supported by the National Cancer Institute (NCI), collecting data from approximately 50% of the US population (16). For both cohorts, patients diagnosed with colon or rectum cancer between 2010 and 2020 who self-identified as NHB or NHW were included. Patients with atypical histologies (e.g., gastrointestinal stromal tumors, leukemia/lymphoma, sarcomas, carcinoid tumors, or mucinous appendiceal neoplasms) or with *in situ* disease, patients missing summary staging information (i.e., local, regional, or distant metastatic disease at diagnosis), or patients missing follow-up data were omitted (Supplementary Figure S1).

### Statistical methods

This study was a retrospective cohort analysis. Descriptive statistics including the chi-square or Kruskal–Wallis tests were used for normally and non-normally distributed data as appropriate. Survival analysis was performed using Cox proportional hazards regression with data visualized using Kaplan–Meier curves. Multivariable Cox regression corrected for sex, geographic region, primary site of disease, and group stage at diagnosis. Survival analyses were further stratified by sex. The primary endpoint was overall survival from the time of diagnosis until censored at the last follow-up; sensitivity analysis was performed using cancer-specific survival (cause of death data available in the SEER). The significance of interaction and stratification was evaluated using the likelihood ratio test. All data results were reported with a point-estimate hazard ratio and a 95% confidence interval, with a two-sided alpha of 0.05 considered significant. All analyses were performed using R version 4.3.1 (17).

## Results

### Patient demographics

The SEER cohort was composed of 226,271 NHW patients (85.7%) and 37,622 NHB patients (14.3%); the VA cohort had 19,515 (80.0) and 4,860 (19.9%), respectively. The VA cohort was more predominantly male patients (>95%) compared to the SEER cohort (52.8%). In the SEER and VA cohorts, NHB patients were more likely than NHW patients to present with distant metastatic disease. In the SEER cohort, the proportions were 28.3% compared to 22.2% (*p* < 0.001), and in the VA cohort, they were 23.0% compared to 19.6% (p < 0.001), respectively. In both cohorts, NHB patients were more likely to present with colorectal cancer at a younger age, specifically under the age of 65 (p < 0.001) (Table 1).

**Table 1:** Patient demographics colorectal cancer diagnosed in 2010–2020.

		SEER Cohort			VA Cohort		
		Non-Hispanic White	Non-Hispanic Black	*p*-value	Non-Hispanic White	Non-Hispanic Black	*p*-value
Overall (%)		226,271 (85.7)	37,622 (14.3)		19,515 (80.0)	4,860 (19.9)	
Age (%)	<50	20,607 (9.1)	4,737 (12.6)	**<0.001**	632 (3.2)	252 (5.2)	**<0.001**
	50–54	19,113 (8.4)	4,229 (11.2)		895 (4.6)	406 (8.4)	
	55–59	21,648 (9.6)	4,934 (13.1)		1,354 (6.9)	687 (14.1)	
	60–64	26,295 (11.6)	5,514 (14.7)		3,278 (16.8)	954 (19.6)	
	65–69	30,133 (13.3)	5,633 (15.0)		4,453 (22.8)	983 (20.2)	
	70–74	29,366 (13.0)	4,424 (11.8)		3,537 (18.1)	633 (13.0)	
	75–79	26,903 (11.9)	3,456 (9.2)		2,157 (11.1)	373 (7.7)	
	80–84	24,731 (10.9)	2,594 (6.9)		1,764 (9.0)	304 (6.3)	
	85+	27,475 (12.1)	2,101 (5.6)		1,445 (7.4)	268 (5.5)	
Sex (%)	Male	120,030 (53.0)	19,292 (51.3)	**<0.001**	18,921 (97.0)	4,646 (95.6)	**<0.001**
Region (%)	West	104,277 (46.1)	10,476 (27.8)	**<0.001**	4,357 (22.3)	564 (11.6)	**<0.001**
	Midwest	15,308 (6.8)	303 (0.8)		5,095 (26.1)	882 (18.1)	
	Northeast	42,042 (18.6)	6,207 (16.5)		2,430 (12.5)	553 (11.4)	
	South	64,644 (28.6)	20,636 (54.9)		7,625 (39.1)	2,858 (58.8)	
Primary site (%)	Rectum	66,477 (29.4)	8,562 (22.8)	**<0.001**	5,242 (26.9)	1,069 (22.0)	**<0.001**
Summary Stage	Localized	88,813 (39.3)	13,484 (35.8)	**<0.001**	10,811 (55.4)	2,477 (51.0)	**<0.001**
	Regional	87,176 (38.5)	13,480 (35.8)		4,877 (25.0)	1,264 (26.0)	
	Distant	50,282 (22.2)	10,658 (28.3)		3,827 (19.6)	1,119 (23.0)	

Patient demographics, stratified by race and grouped by VA and SEER cohorts. VA, Veterans Affairs; SEER, Surveillance, Epidemiology, and End Results; NA, not applicable or missing data. Primary site: rectum versus colon. *p*-values under the pre-specified two-sided threshold of 0.05 are bolded.

### Overall survival in all ages

**Figure 1:**
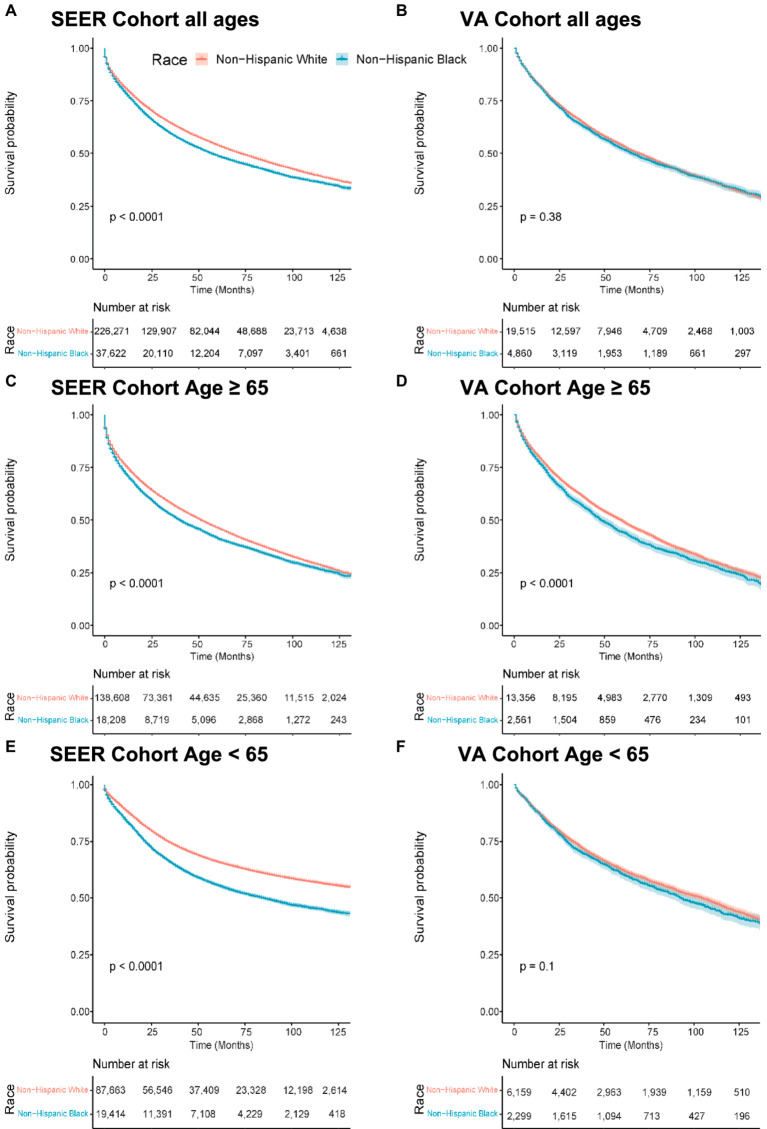
Overall survival by race, Medicare age eligibility, and cohort. Abbreviations: SEER: Surveillance, Epidemiology, and End Results; VA: United States Veterans Health Administration. Kaplan–Meier overall survival curves. **(A)** Patients of all ages from the SEER cohort. **(B)** Patients of all ages from the VA cohort. **(C)** Patients 65 years old and older in the SEER cohort. **(D)** Patients 65 years old and older in the VA cohort. **(E)** Patients less than 65 years old in the SEER cohort. **(F)** Patients less than 65 years old in the VA cohort.

In the SEER cohort, the median overall survival (OS) was 72 months (95% CI 71–73) for NHW patients and 57 months (95% CI 55–58) for NHB patients. In the VA cohort, the median OS was 69 months (95% CI 97–71) for NHW patients compared to 65 months (95% CI 62–69) for NHB patients. Figures 1A,B show the Kaplan–Meier curves visualizing these data. In the SEER cohort, this translated to an NHB versus NHW hazard ratio (HR) of 1.14 (95% confidence interval (CI) 1.12–1.15, *p* = 0.001) compared to the non-significant VA HR of 1.02 (95% CI 0.98–1.07, *p* = 0.375).

After adjusting for age, sex, geographic region, primary site, and grouped stage (i.e., local, regional, or distant disease at diagnosis), the effect size of race increased in both groups. The adjusted OS HR was 1.22 (95% CI 1.20–1.24, *p* < 0.001) in the SEER cohort and 1.05 (95% CI 1.00–1.10, *p* = 0.034) in the VA cohort (Table 2).

**Table 2:** Multivariable Cox regression including all ages in the SEER and VA cohorts.

SEER cohort			
		HR (95% CI)	*p*-value
Race (ref. NHW)	NHB	1.22 (1.2–1.24)	**<0.001**
Age (years, ref. <50)	50–54	1.05 (1.02–1.09)	**0.001**
	55–59	1.3 (1.26–1.34)	**<0.001**
	60–64	1.48 (1.44–1.52)	**<0.001**
	65–69	1.7 (1.65–1.75)	**<0.001**
	70–74	2.15 (2.09–2.21)	**<0.001**
	75–79	2.85 (2.77–2.93)	**<0.001**
	80–84	3.97 (3.87–4.08)	**<0.001**
	≥ 85	6.25 (6.09–6.42)	**<0.001**
Sex (ref. Female)	Male	1.15 (1.14–1.17)	**<0.001**
Region (ref. West)	Midwest	1.02 (1–1.05)	0.063
	Northeast	0.96 (0.95–0.98)	**<0.001**
	South	1.12 (1.11–1.14)	**<0.001**
Primary site (ref Colon)	Rectum	1 (0.98–1.01)	0.68
Grouped stage (ref Local)	Regional	1.57 (1.54–1.59)	**<0.001**
	Distant	7.85 (7.73–7.97)	**<0.001**
VA cohort			
Race (ref NHW)	NHB	1.05 (1.00–1.10)	**0.034**
Age (years, ref. <50)	50–54	0.97 (0.84–1.13)	0.718
	55–59	1.38 (1.22–1.57)	**<0.001**
	60–64	1.49 (1.32–1.68)	**<0.001**
	65–69	1.67 (1.48–1.88)	**<0.001**
	70–74	2.06 (1.83–2.32)	**<0.001**
	75–79	2.81 (2.49–3.18)	**<0.001**
	80–84	3.8 (3.36–4.3)	**<0.001**
	≥ 85	5.56 (4.91–6.3)	**<0.001**
Sex (ref Female)	Male	1.23 (1.09–1.38)	**<0.001**
Region (ref West)	Midwest	1.01 (0.96–1.07)	0.726
	Northeast	0.98 (0.92–1.04)	0.521
	Pacific/other	1.11 (0.8–1.54)	0.518
	South	1.04 (0.99–1.1)	0.09
Primary site (ref Colon)	Rectum	1.22 (1.17–1.27)	**<0.001**
Grouped stage (ref Local)	Regional	1.56 (1.49–1.64)	**<0.001**
	Distant	7.48 (7.16–7.82)	**<0.001**

Multivariable Cox regression for the primary endpoint overall survival. VA, Veterans Affairs; SEER, Surveillance, Epidemiology, and End Results; ref, reference group; HR, hazard ratio, CI, confidence interval. Primary site: rectum versus colon. *p*-values under the pre-specified two-sided threshold of 0.05 are bolded.

### Overall survival stratified by age 65

To study the effect of Medicare age eligibility, subset analyses were conducted of patients ≥65 or < 65 in both the SEER and VA cohorts, with a primary endpoint of OS. In patients ≥65 years of age, NHB patients had worse OS than NHW patients with similar hazard ratios on a univariable analysis: The SEER HR was 1.13 (95% CI 1.11–1.15, *p* < 0.001) and the VA HR was 1.13 (95% CI 1.07–1.19, *p* < 0.001) (Figures 1C,D). A multivariable analysis resulted in the SEER NHB versus NHW HR of 1.17 (95% CI 1.15–1.20, *p* < 0.001), whereas in the VA, the NHB versus NHW HR was attenuated and no longer statistically significant: 1.05 (95% CI 0.99–1.11, *p* = 0.075).

In patients under the age of 65 years in the SEER cohort, NHB compared to NHW had an OS HR of 1.44 (95% CI 1.39–1.49, *p* < 0.001) as compared to the VA with a NHB OS HR of 1.06 (95% CI 0.99–1.14, *p* = 0.10) (Figures 1E,F). In the multivariable analysis, this effect remained significant in the SEER cohort, with NHB versus NHW OS HR of 1.30 (1.27–1.33, *p* < 0.001) (Table 3) but not in the VA: NHB HR 1.05 (95% CI 0.98–1.14, *p* = 0.171). Interaction analysis between ages under 65 years and NHB race in the SEER cohort revealed a combined HR of 1.41 (95% CI 1.34–1.49) and *p* < 0.001 for interaction (likelihood ratio test), whereas in the VA, this revealed an HR of 1.06 (95% CI 0.92–1.23) and *p* = 0.21 for the interaction between race and ages under 65 years.

**Table 3:** Multivariable Cox regression including ages under 65 years in the SEER and VA cohorts.

SEER cohort			
		HR (95% CI)	*p*-value
Race (ref NHW)	NHB	1.30 (1.27–1.33)	**<0.001**
Age (years, ref. <50)	50–54	1.1 (1.06–1.13)	**<0.001**
	55–59	1.34 (1.3–1.38)	**<0.001**
	60–64	1.55 (1.5–1.59)	**<0.001**
Sex (ref Female)	Male	1.2 (1.17–1.22)	**<0.001**
Region (ref West)	Midwest	0.93 (0.88–0.98)	**0.005**
	Northeast	0.93 (0.9–0.96)	**<0.001**
	South	1.13 (1.1–1.15)	**<0.001**
Primary site (ref Colon)	Rectum	0.95 (0.93–0.97)	**<0.001**
Grouped stage (ref Local)	Regional	2.1 (2.03–2.17)	**<0.001**
	Distant	13.21 (12.8–13.64)	**<0.001**
VA cohort			
Race (ref NHW)	NHB	1.05 (0.98–1.14)	0.171
Age (years, ref. <50)	50–54	1.00 (0.86–1.16)	0.995
	55–59	1.43 (1.26–1.63)	**<0.001**
	60–64	1.56 (1.38–1.76)	**<0.001**
Sex (ref Female)	Male	1.22 (1.04–1.43)	**0.017**
Region (ref West)	Midwest	0.98 (0.88–1.09)	0.682
	Northeast	0.97 (0.86–1.1)	0.635
	Pacific/other	0.94 (0.47–1.89)	0.859
	South	1.03 (0.94–1.13)	0.58
Primary site (ref colon)	Rectum	1.26 (1.18–1.35)	**<0.001**
Grouped stage (ref Local)	Regional	1.73 (1.58–1.89)	**<0.001**
	Distant	9.19 (8.48–9.97)	**<0.001**

VA, Veterans Affairs; SEER, Surveillance, Epidemiology, and End Results; NA, not applicable or missing data. Primary site: rectum versus colon. *p*-values under the pre-specified two-sided threshold of 0.05 are bolded.

### Overall survival stratified in patients under 50 years

**Figure 2:**
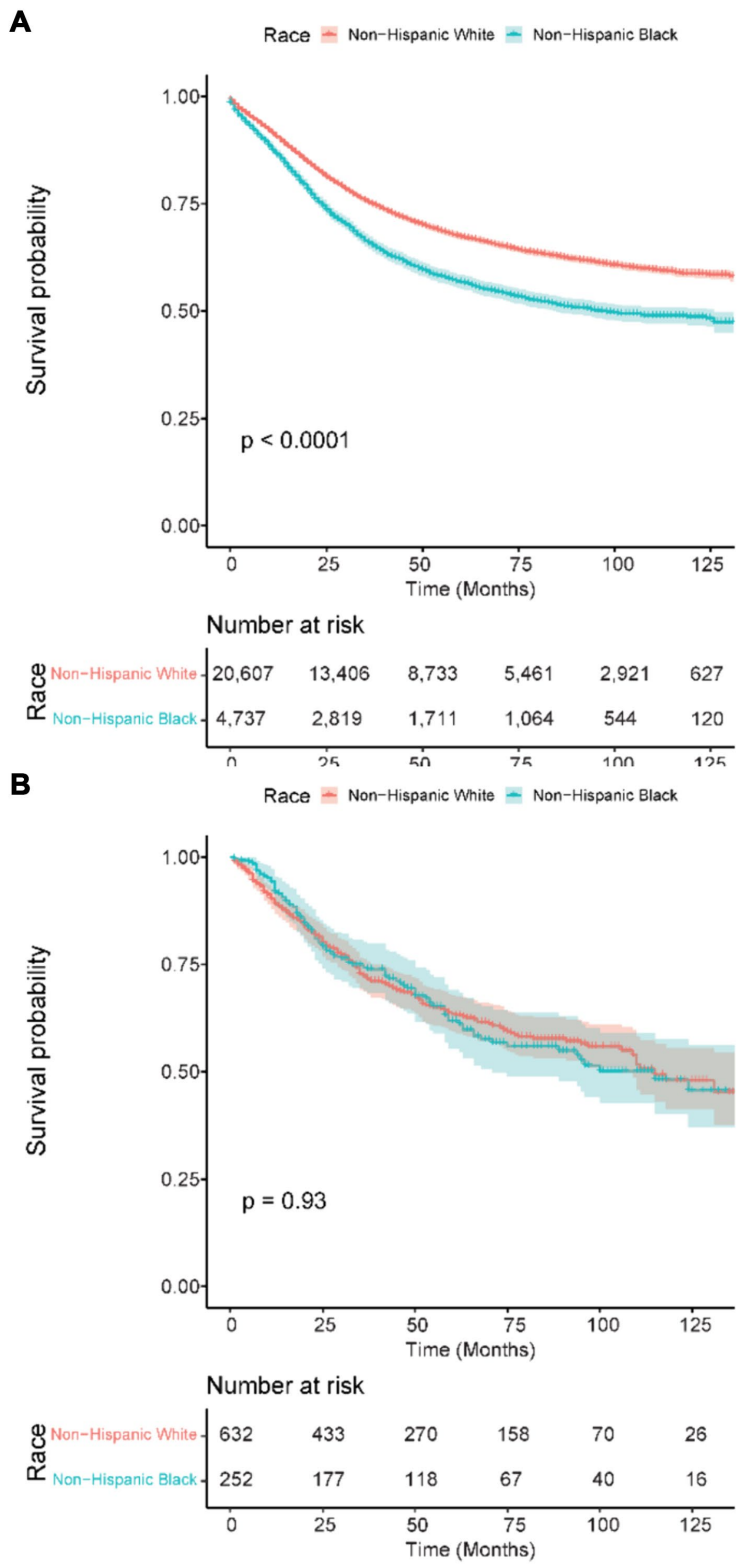
Kaplan–Meier overall survival curves in patients 50 years old and younger. **(A)** SEER patients. **(B)** VA patients. SEER: Surveillance, Epidemiology, and End Results; VA: United States Veterans Health Administration.

A further subset analysis of patients under the age of 50 years found that in the SEER cohort, NHB patients had a lower overall survival, with HR 1.43 (95% CI 1.36–1.51, *p* < 0.001), as compared to the VA where the OS HR for NHB was 1.01 (95% CI 0.79–1.29, *p* = 0.931), visualized in Figure 2. Multivariable analysis (Table 4) found that the effect of the NHB race in the SEER cohort remained significant at HR of 1.35 (95% CI 1.28–1.43, *p* < 0.001) and essentially unchanged in the VA cohort at HR of 1.03 (95% CI 0.80–1.32, *p* = 0.837).

**Table 4:** Multivariable Cox regression including ages under 50 years in the SEER and VA cohorts.

SEER cohort			
		HR (95% CI)	*p*-value
Race (ref NHW)	NHB	1.35 (1.28–1.43)	**<0.001**
Sex (ref Female)	Male	1.19 (1.13–1.24)	**<0.001**
Region (ref West)	Midwest	0.95 (0.86–1.06)	0.393
	Northeast	0.91 (0.86–0.98)	**0.009**
	South	1.09 (1.03–1.14)	**0.001**
Primary site (ref Colon)	Rectum	0.94 (0.9–0.98)	**0.009**
Grouped stage (ref Local)	Regional	2.34 (2.15–2.56)	**<0.001**
	Distant	16.22 (14.92–17.62)	**<0.001**
VA cohort			
Race (ref NHW)	NHB	1.05 (0.81–1.35)	0.729
Sex	Male	1.09 (0.7–1.69)	0.713
Region (ref West)	Midwest	0.88 (0.62–1.25)	0.461
	Northeast	0.95 (0.63–1.45)	0.815
	Pacific/other	3.9 (0.94–16.22)	0.061
	South	0.93 (0.69–1.25)	0.635
Colon/Rectum	Rectum	0.94 (0.74–1.18)	0.576
Grouped Stage	Regional	1.99 (1.39–2.85)	**<0.001**
	Distant	13.19 (9.51–18.28)	**<0.001**

VA, Veterans Affairs; SEER, Surveillance, Epidemiology, and End Results; ref, reference group; HR, hazard ratio, CI, confidence interval. Primary site: rectum versus colon. *p*-values under the pre-specified two-sided threshold of 0.05 are bolded.

### Outcomes stratified by sex

We repeated the overall survival analyses for both cohorts using the grouped and age-stratified approaches but further stratified by sex. Across all ages in the SEER registry, NHB men had an HR of 1.20 (95% CI 1.17–1.22, *p* < 0.001); in comparison, NHB women had an HR of 1.08 (95% CI 1.05–1.10, p < 0.001) compared to NHW women (Supplementary Table S1). Among patients who were 65 years or older, the NHB HR in men was 1.19 (95% CI 1.15–1.22, *p* < 0.001) and NHB women was 1.08 (95% CI 1.05–1.10, *p* < 0.001) (Supplementary Table S2). Among patients under 65 years, NHB men had an HR of 1.44 (95% CI 1.39–1.49, *p* < 0.001) compared to NHW men. In women, the NHB HR was 1.40 (95% CI 1.35–1.49, *p* < 0.001) (Supplementary Table S3). Finally, in patients under 50 years of age, NHB men had an HR of 1.50 (95% CI 1.40–1.61, *p* < 0.001) compared to NHW men, and NHB women had an HR of 1.39 (95% CI 1.28–1.50, *p* < 0.001).

In the VA, across all ages, NHB men had an HR of 1.09 (95% CI 0.99–1.08, *p* = 0.137) compared to NHW men, and NHW women had an HR of 0.77 (95% CI 0.58–1.01, *p* = 0.06) (Supplementary Table S5). In patients aged 65 years and older, NHB men had an HR of 1.13 (95% CI 1.07–1.20, *p* < 0.001) compared to NHW men, and NHB women had an HR of 0.73 (95% CI 0.42–1.24, *p* = 0.242) compared to NHW women (Supplementary Table S6). Under the age of 65 years, the NHB HR was 1.08 (95% CI 1.00–1.16, *p* = 0.055), and in women, it was 0.95 (0.68–1.33, *p* = 0.759) (Supplementary Table S7). Under the age of 50 years, NHB male patients had an HR of 1.02 (95% CI 0.79–1.30, *p* = 0.904) compared to NHW men, and in women, the NHB HR was 0.98 (95% CI 0.40–2.41, *p* = 0.965) compared to NHW women.

### Sensitivity analyses

In the SEER cohort, the above analyses were repeated with cancer-specific mortality as the primary endpoint without any changes to these conclusions (Supplementary Tables S9–S12).

## Discussion

In this analysis, we found that racial disparities between NHB and NHW patients diagnosed with CRC might be linked to equal access to care. In the SEER population, which includes nationwide cancer registry data from patients with all insurance types and a variety of local access to care, there was a highly significant interaction effect between race and age. NHB patients in this cohort aged 65 years and above had an OS HR of 1.13 compared to NHW patients, whereas NHB patients under 65 years had an OS HR of 1.41. This remains significant even after adjusted analyses. Importantly, this trend is not observed in the VA, where patients are insured and typically have equal access to an established care and referral network. While there is still an observed survival difference in the VA, with an OS HR of 1.05 for NHB compared to NHW patients, the magnitude of the NHB/NHW disparity is much smaller than what was observed in the SEER registry and, importantly, there was no effect when stratifying by age of 65 years.

Considering the increasing incidence of early-onset CRC, generally defined as age under 50 years (13), we found results analogous to the other age groups: There was no detectable racial disparity in survival in the VA, whereas there was a large disparity in the SEER population. Our findings suggest equitable access to care with attenuated survival differences between NHB and NHW patients, including in patients who were not typically diagnosed by age-based screening. It should be noted that for this study, we selected a relatively contemporary cohort, but prior to guideline changes for CRC screening, these patients in the <50 years group would likely not have been screening eligible (18–20). This implies that the survival disparity in the SEER cohort is not due to disparate application of cancer screening.

Overall, our findings are consistent with other studies on the benefits of equal-access healthcare systems on racial disparities in CRC. Military health systems have higher rates of uptake of colorectal cancer screening in NHB patients than NHB patients in the general population (possibly higher even than their NHW military/Veteran peers) (21), have equivalent rates of cancer-directed treatment completion (22, 23), and do not face relative delays to receiving definitive treatment (24). However, these findings are not limited to military healthcare systems. For instance, in clinical trials, NHB patients have similar treatment results to NHW patients (11, 25). Recently, data from a cohort of patients enrolled in the Kaiser Permanente Health Insurance Plan (all of whom received care through the same system) found that a systematic CRC screening program was able to reduce NHB/NHW disparities in CRC incidence, late-stage CRC incidence, and CRC death (26). Outside the centralized healthcare systems, programs such as Medicaid expansion following the Affordable Care Act have been associated with a significant improvement in racial disparities in CRC survival (among other malignancies) (27).

It should be noted that in the SEER cohort, we found that among Medicare age-eligible patients, there remained a statistically significant difference in survival in NHB patients, which suggests that insurance barriers alone do not fully explain these disparities. In short, there are several possible explanations for the differences in cancer incidence between NHB and NHW patients, such as dietary/lifestyle risk factors or genetic/epigenetic differences in cancer-related genes (3). However, our study contributes to a body of evidence that racial differences in CRC survival from the time of diagnosis are likely exacerbated by disparate access to care and that policy interventions targeting access to care and screening utilization may attenuate or resolve disparities (while also improving outcomes overall). It remains to be seen whether our findings on racial disparities in equal-access versus non-equal-access settings are the result of better access and quality of care toward health generally (which could, for example, improve patient fitness for surgery, chemotherapy, and radiation) versus cancer-specific care.

There are important limitations to this study. First, while the VA as the comparator cohort offers a large, nationwide, and diverse group of patients, the nature of the US Veteran population (e.g., sex imbalance, military entrance requirements, service-related issues, and post-service resources) may not be generalizable to the entire US population. Additionally, the VA cohort was smaller than the SEER cohort, which may have resulted in less power to detect smaller differences, particularly in the sex-stratified analyses. Nonetheless, the point-estimate hazard ratio in the VA was consistently closer to the null than the SEER cohort in all analyses. In particular, while female patients consistently had better outcomes in both cohorts, the effect of NHB race on survival did not meaningfully differ between men and women, suggesting that sex is not an effect modifier of this NHB/NHW racial disparity. Separately, both databases rely on the accuracy of registrar data collection and entry, and the SEER cohort specifically is limited by the pre-specified variables collected by the program. Consequently, there are finite possibilities for studying possible causal pathways of our findings, and specifically, post-treatment complications are difficult to quantify in terms of the currently available data. Additionally, the retrospective nature of these data limits the ability of this study to draw causal conclusions nor to fully eliminate the risks of unmeasured confounding or other biases that could contribute to the observed results.

As the population of younger people diagnosed with CRC grows, particularly in patients younger than 65 years, efforts to reduce racial disparities in survival outcomes will likely need to address differences in access to care. However, given the higher baseline incidence of CRC in NHB patients (1, 3), closing these survival gaps from the time of diagnosis will likely, on their own, be insufficient to address the overall mortality disparities of CRC. Future studies should further explore causal pathways for racial disparities between these healthcare settings, specifically the timing, completion rate, and rate of complications of each modality of CRC-directed therapies.

## Data availability statement

The data analyzed in this study is subject to the following licenses/restrictions: Data from SEER is available through SEER*Stat for qualified investigators (https://seer.cancer.gov/seerstat/). Data from the VA is available through VINCI for VA investigators with appropriate institutional approval, and is not publicly available due to confidential patient records. Requests to access these datasets should be directed to seer.cancer.gov/seerstat.

## Ethics statement

The studies involving humans were approved by San Diego VA Health Institutional Review Board. The studies were conducted in accordance with the local legislation and institutional requirements. Written informed consent for participation was not required from the participants or the participants’ legal guardians/next of kin in accordance with the national legislation and institutional requirements.

## Author contributions

PR: Conceptualization, Data curation, Formal analysis, Investigation, Methodology, Project administration, Software, Supervision, Validation, Writing – original draft, Writing – review & editing. KM: Data curation, Formal analysis, Methodology, Validation, Writing – original draft, Writing – review & editing. LD: Data curation, Formal analysis, Writing – original draft, Writing – review & editing. JD: Conceptualization, Investigation, Methodology, Writing – original draft, Writing – review & editing. WM: Conceptualization, Investigation, Methodology, Writing – original draft, Writing – review & editing. MM: Investigation, Methodology, Writing – original draft, Writing – review & editing. SG: Conceptualization, Investigation, Writing – original draft, Writing – review & editing. MB: Investigation, Methodology, Writing – original draft, Writing – review & editing. JM: Investigation, Methodology, Supervision, Writing – original draft, Writing – review & editing. BR: Conceptualization, Investigation, Methodology, Project administration, Resources, Supervision, Validation, Writing – original draft, Writing – review & editing.
