# Supplementary material for: Racial disparities in colorectal cancer outcomes and access to care: a multi-cohort analysis
Source: Front Public Health. 2024 Jun 19;12:1414361. doi: 10.3389/fpubh.2024.1414361 (PMC11220245; doi:10.3389/fpubh.2024.1414361)
Supplement: Supplementary file 1 [file Data_Sheet_1.docx]

N = 302,213

NHB or NHW patients with colon or rectal cancer

N = 33,413

SEER Cohort

VA Cohort

N = 21,268

N =5,128

N = 280,945

Omitting atypical histology* or in-situ disease

N = 28,285

N = 16,919

N = 3,253

N = 264,026

Omitting missing staging information

N = 25,032

N = 133

N = 657

N = 263,893

Omitting missing follow up data

N = 24,375

**Supplemental Figure 1: Cohort Selection**

**Supplemental Table 1: SEER patients stratified by sex, all ages**

| **Male Univariable** | | | |
| --- | --- | --- | --- |
|  |  | **HR (95% CI)** | **p value** |
| Race (ref NHW) | NHB | 1.2 (1.17-1.22) | <0.001 |
| **Male Multivariable** | | | |
| Race (ref NHW) | NHB | 1.22 (1.2-1.24) | <0.001 |
| Age (ref <50) | 50-54 | 1.06 (1.03-1.09) | <0.001 |
|  | 55-59 | 1.31 (1.27-1.35) | <0.001 |
|  | 60-64 | 1.49 (1.45-1.53) | <0.001 |
|  | 65-69 | 1.71 (1.66-1.76) | <0.001 |
|  | 70-74 | 2.15 (2.1-2.21) | <0.001 |
|  | 75-79 | 2.84 (2.76-2.91) | <0.001 |
|  | 80-84 | 3.94 (3.83-4.05) | <0.001 |
|  | ≥ 85 | 6.13 (5.97-6.29) | <0.001 |
| Region (ref West) | Midwest | 1.02 (1-1.05) | 0.096 |
|  | Northeast | 0.96 (0.95-0.98) | <0.001 |
|  | South | 1.12 (1.11-1.14) | <0.001 |
| Colon/Rectum | Rectum | 1.01 (0.99-1.02) | 0.261 |
| Grouped Stage (ref local) | Regional | 1.57 (1.54-1.59) | <0.001 |
|  | Distant | 7.86 (7.74-7.97) | <0.001 |
| **Female Univariable** | | | |
|  |  | **HR (95% CI)** | **p value** |
| Race (ref NHW) | NHB | 1.08 (1.05-1.1) | <0.001 |
| **Female Multivariable** | | | |
| Race (ref NHW) | NHB | 1.19 (1.16-1.22) | <0.001 |
| Age (ref <50) | 50-54 | 1.02 (0.97-1.07) | 0.466 |
|  | 55-59 | 1.32 (1.26-1.38) | <0.001 |
|  | 60-64 | 1.48 (1.41-1.54) | <0.001 |
|  | 65-69 | 1.69 (1.62-1.76) | <0.001 |
|  | 70-74 | 2.18 (2.09-2.28) | <0.001 |
|  | 75-79 | 2.84 (2.73-2.96) | <0.001 |
|  | 80-84 | 4.08 (3.92-4.25) | <0.001 |
|  | ≥ 85 | 6.4 (6.16-6.66) | <0.001 |
| Region (ref West) | Midwest | 1.01 (0.98-1.05) | 0.463 |
|  | Northeast | 0.95 (0.93-0.97) | <0.001 |
|  | South | 1.11 (1.09-1.13) | <0.001 |
| Colon/Rectum | Rectum | 0.98 (0.96-1) | 0.017 |
| Grouped Stage (ref local) | Regional | 1.61 (1.57-1.64) | <0.001 |
|  | Distant | 8.23 (8.05-8.41) | <0.001 |

Cox proportional hazards regression studying overall survival in colorectal cancer patients. Abbreviations: SEER = Surveillance, Epidemiology, and End Results; ref = reference group; HR = hazard ratio, CI = confidence interval. Primary site rectum versus colon.

**Supplemental Table 2: SEER patients stratified by sex, age ≥ 65**

| **Male Univariable** | | | |
| --- | --- | --- | --- |
|  |  | **HR (95% CI)** | **p value** |
| Race (ref NHW) | NHB | 1.19 (1.15-1.22) | <0.001 |
| **Male Multivariable** | | | |
| Race (ref NHW) | NHB | 1.22 (1.18-1.26) | <0.001 |
| Age (ref 65-69) | 70-74 | 1.24 (1.2-1.28) | <0.001 |
|  | 75-79 | 1.65 (1.6-1.7) | <0.001 |
|  | 80-84 | 2.24 (2.17-2.31) | <0.001 |
|  | ≥ 85 | 3.48 (3.37-3.59) | <0.001 |
| Region (ref West) | Midwest | 1.08 (1.04-1.12) | <0.001 |
|  | Northeast | 1 (0.97-1.02) | 0.813 |
|  | South | 1.12 (1.1-1.15) | <0.001 |
| Colon/Rectum | Rectum | 1.04 (1.02-1.06) | <0.001 |
| Grouped Stage (ref local) | Regional | 1.43 (1.4-1.46) | <0.001 |
|  | Distant | 6.02 (5.87-6.17) | <0.001 |
| **Female Univariable** | | | |
|  |  | **HR (95% CI)** | **p value** |
| Race (ref NHW) | NHB | 1.08 (1.05-1.10) | <0.001 |
| **Female Multivariable** | | | |
| Race (ref NHW) | NHB | 1.13 (1.09-1.16) | <0.001 |
| Age (ref 65-69) | 70-74 | 1.28 (1.23-1.33) | <0.001 |
|  | 75-79 | 1.66 (1.6-1.72) | <0.001 |
|  | 80-84 | 2.36 (2.28-2.44) | <0.001 |
|  | ≥ 85 | 3.69 (3.57-3.8) | <0.001 |
| Region (ref West) | Midwest | 1.02 (0.98-1.06) | 0.342 |
|  | Northeast | 0.96 (0.94-0.99) | 0.004 |
|  | South | 1.11 (1.08-1.14) | <0.001 |
| Colon/Rectum | Rectum | 1.02 (0.99-1.04) | 0.146 |
| Grouped Stage (ref local) | Regional | 1.51 (1.47-1.55) | <0.001 |
|  | Distant | 6.76 (6.59-6.93) | <0.001 |

Cox proportional hazards regression studying overall survival in colorectal cancer patients. Abbreviations: SEER = Surveillance, Epidemiology, and End Results; ref = reference group; HR = hazard ratio, CI = confidence interval. Primary site rectum versus colon.

**Supplemental Table 3: SEER patients stratified by sex, age < 65**

| **Male Univariable** | | | |
| --- | --- | --- | --- |
|  |  | **HR (95% CI)** | **p value** |
| Race (ref NHW) | NHB | 1.44 (1.39-1.49) | <0.001 |
| **Male Multivariable** | | | |
|  |  | **HR (95% CI)** | **p value** |
| Race (ref NHW) | NHB | 1.30 (1.25-1.34) | <0.001 |
| Age (ref <50) | 50-54 | 1.12 (1.07-1.17) | <0.001 |
|  | 55-59 | 1.33 (1.27-1.38) | <0.001 |
|  | 60-64 | 1.55 (1.49-1.6) | <0.001 |
| Region (ref West) | Midwest | 0.91 (0.85-0.97) | 0.005 |
|  | Northeast | 0.94 (0.9-0.98) | 0.002 |
|  | South | 1.14 (1.1-1.17) | <0.001 |
| Colon/Rectum | Rectum | 0.97 (0.95-1) | 0.072 |
| Grouped Stage (ref local) | Regional | 1.97 (1.89-2.06) | <0.001 |
|  | Distant | 12.18 (11.71-12.68) | <0.001 |
| **Female Univariable** | | | |
|  |  | **HR (95% CI)** | **p value** |
| Race (ref NHW) | NHB | 1.40 (1.35-1.46) | <0.001 |
| **Female Multivariable** | | | |
| Race (ref NHW) | NHB | 1.31 (1.26-1.36) | <0.001 |
| Age (ref <50) | 50-54 | 1.06 (1.01-1.12) | 0.024 |
|  | 55-59 | 1.37 (1.31-1.44) | <0.001 |
|  | 60-64 | 1.55 (1.48-1.62) | <0.001 |
| Region (ref West) | Midwest | 0.96 (0.88-1.05) | 0.358 |
|  | Northeast | 0.92 (0.88-0.97) | 0.001 |
|  | South | 1.11 (1.07-1.15) | <0.001 |
| Colon/Rectum | Rectum | 0.92 (0.88-0.95) | <0.001 |
| Grouped Stage (ref local) | Regional | 2.33 (2.2-2.46) | <0.001 |
|  | Distant | 15.11 (14.34-15.93) | <0.001 |

Cox proportional hazards regression studying overall survival in colorectal cancer patients. Abbreviations: VA = Veterans Affairs; SEER = Surveillance, Epidemiology, and End Results; ref = reference group; HR = hazard ratio, CI = confidence interval. Primary site rectum versus colon.

**Supplemental Table 4: SEER patients stratified by sex, age < 50**

| **Male Univariable** | | | |
| --- | --- | --- | --- |
|  |  | **HR (95% CI)** | **p value** |
| Race (ref NHW) | NHB | 1.50 (1.40-1.61) | <0.001 |
| **Male Multivariable** | | | |
|  |  | **HR (95% CI)** | **p value** |
| Race (ref NHW) | NHB | 1.35 (1.25-1.45) | <0.001 |
| Region (ref West) | Midwest | 0.92 (0.8-1.06) | 0.241 |
|  | Northeast | 0.92 (0.85-1.01) | 0.074 |
|  | South | 1.09 (1.02-1.17) | 0.011 |
| Colon/Rectum | Rectum | 0.98 (0.93-1.05) | 0.613 |
| Grouped Stage (ref local) | Regional | 2.12 (1.9-2.38) | <0.001 |
|  | Distant | 13.92 (12.51-15.49) | <0.001 |
| **Female Univariable** | | | |
|  |  | **HR (95% CI)** | **p value** |
| Race (ref NHW) | NHB | 1.39 (1.28-1.5) | <0.001 |
| **Female Multivariable** | | | |
| Race (ref NHW) | NHB | 1.36 (1.25-1.47) | <0.001 |
| Region (ref West) | Midwest | 1.01 (0.85-1.19) | 0.922 |
|  | Northeast | 0.9 (0.81-1) | 0.051 |
|  | South | 1.08 (1-1.17) | 0.049 |
| Colon/Rectum | Rectum | 0.88 (0.82-0.95) | <0.001 |
| Grouped Stage (ref local) | Regional | 2.67 (2.32-3.08) | <0.001 |
|  | Distant | 19.87 (17.41-22.68) | <0.001 |

Cox proportional hazards regression studying overall survival in colorectal cancer patients. Abbreviations: VA = Veterans Affairs; SEER = Surveillance, Epidemiology, and End Results; ref = reference group; HR = hazard ratio, CI = confidence interval. Primary site rectum versus colon.

**Supplemental Table 5: VA patients stratified by sex, all ages**

| **Male Univariable** | | | |
| --- | --- | --- | --- |
|  |  | HR (95% CI) | p-value |
| Race (ref NHW) | NHB | 1.03 (0.99-1.08) | 0.137 |
| **Male Multivariable** | | | |
| Race (ref NHW) | NHB | 1.06 (1.01-1.11) | **0.012** |
| Age (ref <50) | 50-54 | 0.96 (0.83-1.12) | 0.618 |
|  | 55-59 | 1.39 (1.22-1.59) | **<0.001** |
|  | 60-64 | 1.49 (1.32-1.68) | **<0.001** |
|  | 65-69 | 1.66 (1.47-1.88) | **<0.001** |
|  | 70-74 | 2.06 (1.82-2.33) | **<0.001** |
|  | 75-79 | 2.8 (2.47-3.18) | **<0.001** |
|  | 80-84 | 3.76 (3.31-4.27) | **<0.001** |
|  | ≥ 85 | 5.56 (4.89-6.32) | **<0.001** |
| Colon/Rectum | Rectum | 1.22 (1.17-1.27) | **<0.001** |
| Grouped Stage | Regional | 1.56 (1.49-1.63) | **<0.001** |
|  | Distant | 7.43 (7.1-7.77) | **<0.001** |
| **Female Univariable** | | | |
|  |  | HR (95% CI) | p-value |
| Race (ref NHW) | NHB | 0.77 (0.58-1.01) | 0.06 |
| **Female Multivariable** | | | |
| Race (ref NHW) | NHB | 1.01 (0.75-1.35) | 0.974 |
| Age (ref <50) | 50-54 | 1.15 (0.66-1.98) | 0.624 |
|  | 55-59 | 1.3 (0.78-2.16) | 0.317 |
|  | 60-64 | 1.41 (0.86-2.32) | 0.174 |
|  | 65-69 | 1.53 (0.89-2.62) | 0.12 |
|  | 70-74 | 1.94 (1.11-3.37) | **0.019** |
|  | 75-79 | 3.17 (1.75-5.75) | **<0.001** |
|  | 80-84 | 5.71 (3.05-10.69) | **<0.001** |
|  | ≥ 85 | 4.05 (2.23-7.37) | **<0.001** |
| Colon/Rectum | Rectum | 0.95 (0.71-1.27) | 0.735 |
| Grouped Stage | Regional | 1.59 (1.16-2.17) | **0.004** |
|  | Distant | 9.45 (7.11-12.55) | **<0.001** |

Cox proportional hazards regression studying overall survival in colorectal cancer patients. Note that due to low patient counts, region was not included in this gender-stratified analysis. Abbreviations: VA = Veterans Affairs; ref = reference group; HR = hazard ratio, CI = confidence interval. Primary site rectum versus colon.

**Supplemental Table 6: VA patients stratified by sex, age ≥ 65**

| **Male Univariable** | | | |
| --- | --- | --- | --- |
|  |  | HR (95% CI) | p-value |
| Race (ref NHW) | NHB | 1.13 (1.07-1.2) | **<0.001** |
| **Male Multivariable** | | | |
| Race (ref NHW) | NHB | 1.06 (1-1.12) | **0.041** |
| Age (ref 65-69) | 70-74 | 1.24 (1.17-1.32) | **<0.001** |
|  | 75-79 | 1.67 (1.57-1.79) | **<0.001** |
|  | 80-84 | 2.25 (2.1-2.4) | **<0.001** |
|  | ≥ 85 | 3.32 (3.1-3.56) | **<0.001** |
| Colon/Rectum | Rectum | 1.2 (1.14-1.26) | **<0.001** |
| Grouped Stage | Regional | 1.51 (1.43-1.6) | **<0.001** |
|  | Distant | 6.85 (6.5-7.23) | **<0.001** |
| **Female Univariable** | | | |
|  |  | HR (95% CI) | p-value |
| Race (ref NHW) | NHB | 0.73 (0.42-1.24) | 0.242 |
| **Female Multivariable** | | | |
| Race (ref NHW) | NHB | 0.90 (0.52-1.57) | 0.713 |
| Age (ref 65-69) | 70-74 | 1.21 (0.74-1.96) | 0.448 |
|  | 75-79 | 1.82 (1.06-3.11) | **0.03** |
|  | 80-84 | 3.23 (1.82-5.74) | **<0.001** |
|  | ≥ 85 | 2.6 (1.53-4.44) | **<0.001** |
| Colon/Rectum | Rectum | 1 (0.63-1.59) | 0.989 |
| Grouped Stage | Regional | 1.44 (0.93-2.24) | 0.104 |
|  | Distant | 5.06 (3.3-7.76) | **<0.001** |

Cox proportional hazards regression studying overall survival in colorectal cancer patients. Note that due to low patient counts, region was not included in this gender-stratified analysis. Abbreviations: VA = Veterans Affairs; SEER = Surveillance, Epidemiology, and End Results; ref = reference group; HR = hazard ratio, CI = confidence interval. Primary site rectum versus colon.

**Supplemental Table 7: VA patients stratified by sex, age < 65**

| **Male Univariable** | | | |
| --- | --- | --- | --- |
|  |  | HR (95% CI) | p-value |
| Race (ref NHW) | NHB | 1.08 (1.00-1.16) | 0.055 |
| **Male Multivariable** | | | |
| Race (ref NHW) | NHB | 1.07 (0.99-1.15) | 0.096 |
| Age (ref <50) | 50-54 | 0.99 (0.85-1.15) | 0.886 |
|  | 55-59 | 1.44 (1.26-1.65) | **<0.001** |
|  | 60-64 | 1.56 (1.38-1.77) | **<0.001** |
| Colon/Rectum | Rectum | 1.27 (1.19-1.37) | **<0.001** |
| Grouped Stage | Regional | 1.72 (1.57 -1.88) | **<0.001** |
|  | Distant | 8.93 (8.22-9.69) | **<0.001** |
| **Female Univariable** | | | |
|  |  | HR (95% CI) | p-value |
| Race (ref NHW) | NHB | 0.95 (0.68-1.33) | 0.759 |
| **Female Multivariable** | | | |
| Race (ref NHW) | NHB | 1.10 (0.78-1.56) | 0.584 |
| Age (ref <50) | 50-54 | 1.12 (0.64-1.93) | 0.698 |
|  | 55-59 | 1.21 (0.72-2.03) | 0.465 |
|  | 60-64 | 1.30 (0.79-2.16) | 0.304 |
| Colon/Rectum | Rectum | 0.99 (0.68-1.45) | 0.963 |
| Grouped Stage | Regional | 1.81 (1.15-2.85) | **0.010** |
|  | Distant | 17.09 (11.35-25.74) | **<0.001** |

Cox proportional hazards regression studying overall survival in colorectal cancer patients. Note that due to low patient counts, region was not included in this gender-stratified analysis. Abbreviations: VA = Veterans Affairs; SEER = Surveillance, Epidemiology, and End Results; ref = reference group; HR = hazard ratio, CI = confidence interval. Primary site rectum versus colon.

**Supplemental Table 8: VA patients stratified by sex, age < 50**

| **Male Univariable** | | | |
| --- | --- | --- | --- |
|  |  | HR (95% CI) | p-value |
| Race (ref NHW) | NHB | 1.02 (0.79-1.3) | 0.904 |
| **Male Multivariable** | | | |
| Race (ref NHW) | NHB | 1.06 (0.82-1.37) | 0.648 |
| Colon/Rectum | Rectum | 0.94 (0.74-1.2) | 0.623 |
| Grouped Stage | Regional | 1.88 (1.31-2.72) | <0.001 |
|  | Distant | 11.27 (8.1-15.67) | <0.001 |
| **Female Univariable** | | | |
|  |  | HR (95% CI) | p-value |
| Race (ref NHW) | NHB | 0.98 (0.4-2.41) | 0.965 |
| **Female Multivariable** | | | |
| Race (ref NHW) | NHB | 1.00 (0.38-2.65) | 0.997 |
| Age (ref <50) | 50-54 | 0.70 (0.24-2.07) | 0.517 |
|  | 55-59 | 2.28 (0.51-10.28) | 0.282 |
|  | 60-64 | 337.9 (33.07-3452.01) | <0.001 |
| Colon/Rectum | Rectum | 1.00 (0.38-2.65) | 0.997 |
| Grouped Stage | Regional | 0.70 (0.24-2.07) | 0.517 |
|  | Distant | 2.28 (0.51-10.28) | 0.282 |

Cox proportional hazards regression studying overall survival in colorectal cancer patients. Note that due to low patient counts, region was not included in this gender-stratified analysis. Abbreviations: VA = Veterans Affairs; SEER = Surveillance, Epidemiology, and End Results; ref = reference group; HR = hazard ratio, CI = confidence interval. Primary site rectum versus colon.

**Supplemental Table 9: SEER analysis of cancer-specific death, all ages**

| **Univariable** | | | |
| --- | --- | --- | --- |
|  |  | HR (95% CI) | p-value |
| Race (ref NHW) | NHB | 1.23 (1.2-1.25) | <0.001 |
| **Multivariable** | | | |
| Race (ref NHW) | NHB | 1.23 (1.2-1.25) | <0.001 |
| Age (ref <50) | 50-54 | 1.04 (1.01-1.08) | 0.016 |
|  | 55-59 | 1.22 (1.18-1.26) | <0.001 |
|  | 60-64 | 1.34 (1.3-1.38) | <0.001 |
|  | 65-69 | 1.43 (1.39-1.47) | <0.001 |
|  | 70-74 | 1.7 (1.65-1.75) | <0.001 |
|  | 75-79 | 2.1 (2.03-2.16) | <0.001 |
|  | 80-84 | 2.79 (2.7-2.88) | <0.001 |
|  | ≥ 85 | 4.32 (4.2-4.46) | <0.001 |
| Sex (ref Female) | Male | 1.08 (1.06-1.1) | <0.001 |
| Region (ref West) | Midwest | 0.99 (0.96-1.02) | 0.633 |
|  | Northeast | 0.9 (0.88-0.92) | <0.001 |
|  | South | 1.07 (1.05-1.09) | <0.001 |
| Colon/Rectum | Rectum | 1.03 (1.02-1.05) | <0.001 |
| Grouped Stage | Regional | 2.68 (2.62-2.74) | <0.001 |
|  | Distant | 17.24 (16.87-17.62) | <0.001 |

Cox proportional hazards regression studying cancer specific survival in colorectal cancer patients. Abbreviations: SEER = Surveillance, Epidemiology, and End Results; ref = reference group; HR = hazard ratio, CI = confidence interval. Primary site rectum versus colon.

**Supplemental Table 10: SEER analysis of cancer-specific death, age ≥ 65**

| **Univariable** | | | |
| --- | --- | --- | --- |
|  |  | HR (95% CI) | p-value |
| Race (ref NHW) | NHB | 1.20 (1.17-1.23) | <0.001 |
| **Multivariable** | | | |
| Race (ref NHW) | NHB | 1.18 (1.15-1.21) | <0.001 |
| Age (ref 65-69) | 70-74 | 1.18 (1.14-1.21) | <0.001 |
|  | 75-79 | 1.44 (1.4-1.48) | <0.001 |
|  | 80-84 | 1.89 (1.84-1.94) | <0.001 |
|  | ≥ 85 | 2.86 (2.79-2.94) | <0.001 |
| Sex (ref Female) | Male | 1.04 (1.03-1.06) | <0.001 |
| Region (ref West) | Midwest | 1.02 (0.99-1.06) | 0.233 |
|  | Northeast | 0.9 (0.88-0.92) | <0.001 |
|  | South | 1.06 (1.03-1.08) | <0.001 |
| Colon/Rectum | Rectum | 1.09 (1.07-1.11) | <0.001 |
| Grouped Stage | Regional | 2.47 (2.41-2.54) | <0.001 |
|  | Distant | 14.19 (13.83-14.55) | <0.001 |

Cox proportional hazards regression studying cancer specific survival in colorectal cancer patients. Abbreviations: SEER = Surveillance, Epidemiology, and End Results; ref = reference group; HR = hazard ratio, CI = confidence interval. Primary site rectum versus colon.

**Supplemental Table 11: SEER analysis of cancer-specific death, age < 65**

| **Univariable** | | | |
| --- | --- | --- | --- |
|  |  | HR (95% CI) | p-value |
| Race (ref NHW) | NHB | 1.4 (1.36-1.44) | <0.001 |
| **Multivariable** | | | |
| Race (ref NHW) | NHB | 1.28 (1.25-1.32) | <0.001 |
| Age (ref <50) | 50-54 | 1.06 (1.02-1.1) | 0.001 |
|  | 55-59 | 1.24 (1.2-1.29) | <0.001 |
|  | 60-64 | 1.38 (1.33-1.42) | <0.001 |
| Sex (ref Female) | Male | 1.15 (1.12-1.18) | <0.001 |
| Region (ref West) | Midwest | 0.91 (0.86-0.97) | 0.002 |
|  | Northeast | 0.9 (0.87-0.93) | <0.001 |
|  | South | 1.08 (1.05-1.11) | <0.001 |
| Colon/Rectum | Rectum | 0.96 (0.94-0.99) | 0.002 |
| Grouped Stage | Regional | 3.42 (3.27-3.59) | <0.001 |
|  | Distant | 25.57 (24.48-26.71) | <0.001 |

Cox proportional hazards regression studying cancer specific survival in colorectal cancer patients. Abbreviations: SEER = Surveillance, Epidemiology, and End Results; ref = reference group; HR = hazard ratio, CI = confidence interval. Primary site rectum versus colon.

**Supplemental Table 12: SEER analysis of cancer-specific death, age < 50**

| **Univariable** | | | |
| --- | --- | --- | --- |
|  |  | HR (95% CI) | p-value |
| Race (ref NHW) | NHB | 1.42 (1.34-1.5) | <0.001 |
| **Multivariable** | | | |
| Race (ref NHW) | NHB | 1.35 (1.27-1.43) | <0.001 |
| Sex (ref Female) | Male | 1.18 (1.13-1.24) | <0.001 |
| Region (ref West) | Midwest | 0.94 (0.83-1.05) | 0.26 |
|  | Northeast | 0.9 (0.84-0.97) | 0.004 |
|  | South | 1.06 (1-1.12) | 0.043 |
| Colon/Rectum | Rectum | 0.95 (0.91-1) | 0.057 |
| Grouped Stage | Regional | 3.18 (2.86-3.55) | <0.001 |
|  | Distant | 24.12 (21.77-26.73) | <0.001 |

Cox proportional hazards regression studying cancer specific survival in colorectal cancer patients. Abbreviations: SEER = Surveillance, Epidemiology, and End Results; ref = reference group; HR = hazard ratio, CI = confidence interval. Primary site rectum versus colon.
